# RNA modifications in brain tumorigenesis

**DOI:** 10.1186/s40478-020-00941-6

**Published:** 2020-05-06

**Authors:** Albert Z. Huang, Alberto Delaidelli, Poul H. Sorensen

**Affiliations:** 1 Department of Molecular Oncology, British Columbia Cancer Research Centre, Vancouver, BC V5Z 1L3 Canada; 2 Department of Pathology and Laboratory Medicine, University of British Columbia, Vancouver, BC V6T 1Z3 Canada

**Keywords:** Brain tumors, mRNA modifications, Glioma, Post-translational modifications, Alternative splicing, Alternative polyadenylation (APA), Inosine, *N*^6^-methyladenosine (m^6^A)

## Abstract

RNA modifications are emerging as critical regulators in cancer biology, thanks to their ability to influence gene expression and the predominant protein isoforms expressed during cell proliferation, migration, and other pro-oncogenic properties. The reversibility and dynamic nature of post-transcriptional RNA modifications allow cells to quickly adapt to microenvironmental changes. Recent literature has revealed that the deregulation of RNA modifications can promote a plethora of developmental diseases, including tumorigenesis. In this review, we will focus on four key post-transcriptional RNA modifications which have been identified as contributors to the pathogenesis of brain tumors: m^6^A, alternative polyadenylation, alternative splicing and adenosine to inosine modifications. In addition to the role of RNA modifications in brain tumor progression, we will also discuss potential opportunities to target these processes to improve the dismal prognosis for brain tumors.

## Introduction

Of the approximately 25,000 people diagnosed annually with primary malignant brain tumors in the USA, 80% are gliomas, one of the most lethal types of cancer [89, 101, 131]. Amongst gliomas, glioblastoma (GBM) is the most aggressive, characterized by a median patient survival of less than 15 months following surgical resection and concurrent radiotherapy and chemotherapy with temozolomide (TMZ) [67, 111]. Most of the literature on gliomas has historically focused on transcriptional control of gene expression [81, 124]. However, the role of post-transcriptional RNA modifications in cellular function and glioma progression has recently begun to surface, mostly due to advances in next generation sequencing (NGS) [46, 115, 145]. The insights gained from these advances highlight the importance of post-transcriptional control of gene expression in the development and progression of brain tumors as well as neurological disorders such as autism, Alzheimer’s disease, and Parkinson’s disease [21, 22, 51, 62].

Before messenger RNA (mRNA) translation and protein synthesis can occur, nascent mRNA transcripts require processing and nucleic acid modifications. These include splicing out of introns, the non-coding sections of the transcripts, as well as methylation of certain bases. RNA modifications modulate most steps of gene expression, from indirectly controlling DNA transcription, by regulating expression of mRNAs encoding transcription factors, to directly affecting mRNA translation [23, 144]. While many RNA modifications were originally discovered decades ago [24, 92], studies of many of these modifications, such as N^6^-methyladenosine (m^6^A), were previously limited by the inability to distinguish between certain nucleotides during reverse transcription [20, 96]. However, with the recent advances in NGS, over one hundred different types of RNA modifications have now been described [8, 11, 65, 69]. Other RNA modifications include N^1^-methyladenosine (m^1^A) and 5-methylcytosine (m^5^C), which are not found only in mRNA: m^5^C can also be found in transfer RNA (tRNA) and m^1^A in tRNAs, ribosomal RNAs (rRNAs) and long non-coding RNAs (lncRNAs) [25, 96]. Within rRNAs, the most abundant modification are 2′-O-methylations, which are known to play a significant role in ribosome function [30]. These modifications are not further discussed here and are reviewed elsewhere [96], as their roles in brain tumorigenesis are not well established. For the purpose of this review, we will focus on the most common RNA modifications that have been characterized in glioma: m^6^A, alternative polyadenylation (APA), alternative splicing, and adenosine to inosine (A-to-I) modifications. RNA modifications are a highly conserved mechanism utilized by eukaryotes throughout phylogeny to enhance biologic complexity [7]. RNA modifications are typically both reversible and dynamic, allowing for rapid cellular adaptation to changes in the microenvironment, thus limiting the size of the genome necessary to encode adaptive molecules [6, 28]. This mechanism is particularly beneficial for cancer cells to adapt to acute microenvironmental changes [6, 28]. In contrast to the relatively long half-life of mRNA in mammalian cells (median of 9 h) through protracted transcriptional changes, dynamic RNA alterations can be completed in < 30 s [12]. This allows for rapid cellular adaptation to harsh environments, most commonly induced in cancer by microenvironmental stresses, such as hypoxia, or toxic therapy [23, 100]. While several informative publications have underscored the pivotal role of RNA modifications in glioma progression [79, 84], a comprehensive overview on the topic is currently lacking. We believe that a better understanding of the molecular mechanisms behind glioma progression is critical for the development of novel therapeutic approaches that could ultimately improve the outcome of patients with glioma.

## *N*^6^-methyladenosine (m^6^A) modifications

Of the known RNA modifications, the methylation of adenosine at the nitrogen-6 position to create *N*^6^-methyladenosine (m^6^A) makes up the majority of the internal mRNA modifications in eukaryotes, and has emerged as a critical regulator in many aspects of RNA biology, including pre-mRNA splicing, polyadenylation, localization, and mRNA translation [23, 27, 95]. The addition, removal and recognition of m^6^A is catalyzed by methyltransferases, demethylases, and binding proteins, otherwise known as “writers,” “erasers” and “readers”, respectively [82]. M^6^A generally occurs within long exons, around stop codons, and in 3′ untranslated regions (3′-UTRs) [140, 141]. However, approximately 30% of target sites for m^6^A writers are also located in intronic RNA regions [69], indicating that m^6^A methylation may occur co-transcriptionally, before or during splicing. In addition, mRNA splicing factor precursors co-localize with m^6^A methyltransferases in nuclear speckles, suggesting the involvement of intronic m^6^A residues in alternative splicing [61, 69].

The relative ease by which m^6^A can be added or removed facilitates rapid changes in gene expression, as m^6^A modifications are reported to promote mRNA decay through binding of specific degradative protein complexes [83, 140]. This is in contrast to the historical notion that RNA molecules remain largely unchanged after initial covalent modifications [33]. To catalyze m^6^A mRNA methylation, the multi-subunit writer complex comprises a catalytic subunit, known as methyltransferase-like 3 (METTL3), a second augmenting methyltransferase subunit (METTL14) utilized in substrate recognition, as well as the Wilms’ tumor 1-associating protein (WTAP) [9, 82, 127]. Lacking the methyltransferase activity of the other subunits, WTAP is instead likely to be involved in m^6^A modifications by promoting the recruitment of the METTL3-METTL14 complex to target mRNAs, in addition to inducing the translocation of the complex to nuclear speckles [68, 69]. WTAP overexpression promotes the migratory and invasive capabilities of GBM cells by epidermal growth factor receptor (EGFR) stimulation, although no further mechanistic insights were provided in this study [54]. *WTAP* mutations are extremely rare in cancer, occurring in only 0.5% of gliomas [13]. Instead, utilizing Quaking gene isoform 6 (QKI-6) knockout and QKI-6 mutant studies in glioma U87 and U251 cell lines, and tissues derived from GBM, Xi et al. found that WTAP is regulated by QKI-6. WTAP mRNAs contain a specific sequence known as a QKI response element (QRE) in its 3′ UTR region whereby QKI-6 induces WTAP expression [134]. Moreover, QKI-6 is directly controlled by microRNAs (miRNAs), with miR-29a overexpression leading to reduced QKI-6 activity and decreased glioma tumor growth and increased survival [134]. While further studies are required to fully elucidate the mechanism behind WTAP function in GBM pathogenesis, it is reasonable to postulate that WTAP’s activity of recruiting methyltransferases to specific unidentified targets facilitates GBM progression, and the utilization of miRNA-based therapies could prove beneficial for glioma treatment.

Proteins often referred to as “m^6^A readers” selectively bind to mRNAs modified with m^6^A. The specific type of reader protein regulates different functions: binding of YTH domain containing family protein (YTHDC1) to m^6^A induces mRNA splicing by recruiting splicing factor SRSF3 [135], whereas binding of YTHDF2 targets the transcripts for degradation by recruiting them to cytoplasmic processing (P) bodies within mammalian cells [29, 50, 128]. In contrast, transcript binding by YTHDF1 and YTHDF3 enhances their translation [70, 128, 129]. To facilitate transcript binding, a hydrophobic pocket within the YTH domain interacts with the methyl group exposed in m^6^A [64, 70, 117, 136]. While *YTHDF1* and *YTHDF2* mutations only occur in 0.9 and 0.5% of glioma cases respectively [13], several published datasets, including from The Cancer Genome Atlas (TCGA), show that YTHDF1 and YTHDF2 mRNA expression levels are positively correlated with malignancy of gliomas, with significant increases in higher grade gliomas, suggesting a role for these m^6^A readers in glioma progression [13, 15, 112]. While this seems counterintuitive at first glance, given the different effects on mRNAs by binding these proteins, one possible explanation is provided by Wang et al. [129]. Using photoactivatable ribonucleoside-enhanced crosslinking and immunoprecipitation (PAR-CLIP) and RNA immunoprecipiation (RIP-seq), the authors identified 1260 and 1276 mRNA targets for YTHDF1 and YTHDF2, respectively [129]. While these proteins share 622 mRNA targets, YTHDF1 and YTHDF2 also bind to ~ 650 unique mRNA targets each [129]. Although this experiment was performed in human cervical cancer HeLa cells, unique regulation of separate mRNAs by YTHDF1 versus YTHDF2 in gliomas could provide an intriguing explanation for overexpression of both genes in high grade gliomas. For example, YTHDF1 could drive translation of pro-oncogenic transcripts, while YTHDF2 might drive degradation of tumor suppressor encoding transcripts. While YTHDF1 and YTHDF2 expression promote pancreatic and lung cancer cell proliferation, no equivalent research has to date determined a causal relationship between YTHDF1 or YTHDF2 expression in gliomagenesis [17, 104, 105]. It also remains to be determined if YTHDF1 and/or YTHDF3 are upregulated epigenetically in gliomas.

The reversal of m^6^A methylation is catalyzed by demethylases known as fat mass and obesity-associated protein (FTO), and ALKBH5 [125, 146], both acting as so-called “erasers” for m^6^A modifications [53, 146]. In glioma, mutations occur only in 0.1% of cases for *ALKBH5* and no mutations have been reported in *FTO* [13]. However, as mentioned earlier, high expression of ALKBH5, which could occur through the induction of hypoxia-inducible factors (HIFs), as seen in breast cancer [142], is linked to worse GBM patient outcome [139, 143]. To further investigate the mechanism, Zhang et al. immunoprecipitated RNAs using m^6^A primary antibodies and performed microarray analysis. This approach identified ALKBH5 mRNAs targets, such as the proto-oncogene *FOXM1.* Demethylation of m^6^A residues in the 3′-UTR of the FOXM1 pre-mRNA results in increased transcript stability and enhanced FOXM1 protein expression [143]. This leads to downstream STAT3 activation and thus increased GBM proliferation, invasion and metastasis [39]. Demethylation of mRNA increases binding of the RNA stabilizer protein Hu-antigen R (HUR), and thus leads to increased stability of the targeted mRNA [83].

According to the TCGA, genetic amplifications at the *METTL3* locus arise in ~ 1% of gliomas [13]. In addition, several studies have shown that METTL3 mRNA and m^6^A levels are elevated in glioma compared to normal brain [19, 112, 125], therefore leading multiple researchers to investigate the effects of upregulating and suppressing METTL3 on glioma growth. Early research indicated that short hairpin RNA (shRNA) mediated silencing of METTL3 in several glioma cell lines, both in vitro as well as in in vivo orthotopic models, resulted in enhanced GBM growth [19]. As a possible explanation of this phenotype, the authors found by RNA-seq that oncogenes such as *ADAM19* and *KLF4* were upregulated by METTL3 silencing and tumor suppressors such as *CDKN2A* and *BRCA2* were downregulated. However, more recent work suggests a different scenario. Visvanathan et al., utilizing methylated RNA immunoprecipitation-seq (MeRIP-seq) on glioma cell line MGG8, followed by gene set enrichment (GSEA) and Gene Ontology (GO) analyses, proposed that METTL3 silencing may in fact disrupt tumorigenic pathways that facilitate glioma progression, such as NOTCH, c-Myc and NFκB [63, 125]. This concept was further supported by Li et al., who found that both genetic knockout and knockdown of *METTL3 *significantly decreased proliferation of GBM cell lines U251 and U87MG in cell viability assays [63]. In vivo, xenograft tumor size was reduced compared to controls after inoculation of shMETTL3 GBM cells into mice [63]. While it is difficult to speculate on these inconsistencies, it is possible that some of the observed differences could be due to intertumor heterogeneity and the use of different cell lines [125]. The METTL3-METTL14 complex shares similar structures with other DNA and protein methyltransferases, including disrupter of telomeric silencing 1-like (DOT1L), as both contain Rossmann fold structural motifs [99, 108]. Notably, small-molecule inhibitors of DOT1L are currently undergoing clinical trials to treat acute myeloid leukemia (AML), suggesting the potential to develop novel drug therapies targeting against this family of proteins in brain tumors [110].

Additional m^6^A methylation regulators are also emerging as critical components of GBM tumorigenesis [19, 126]. Chai et al. reported that many of the main regulators of m^6^A modifications are differentially expressed between different glioma grades. Specifically, there is positive correlation between WHO grade and expression of WTAP, YT521-B homology (YTH) domain containing family (YTHDF) and AlkB homolog 5 (ALKBH5), whereas there is a negative correlation between FTO and WHO grade [15]. These results suggest a potential interplay among these regulatory elements and glioma malignancy [15]. A possible mechanism of action for these enzymes in the context of glioma progression was recently proposed by Li et al. The authors suggest that METTL3 is involved in decreasing nonsense-mediated mRNA decay (NMD) of transcripts encoding for splicing factors by m^6^A deposition around the start codon of serine and arginine rich splicing factor (SRSF) mRNAs. The methylation around the start codon then prevents NMD of SRSF mRNAs, thus resulting in increased alternative splicing and isoform switching in glioma [63].

## Alternative 3′ polyadenylation (APA)

**Fig. 1:**
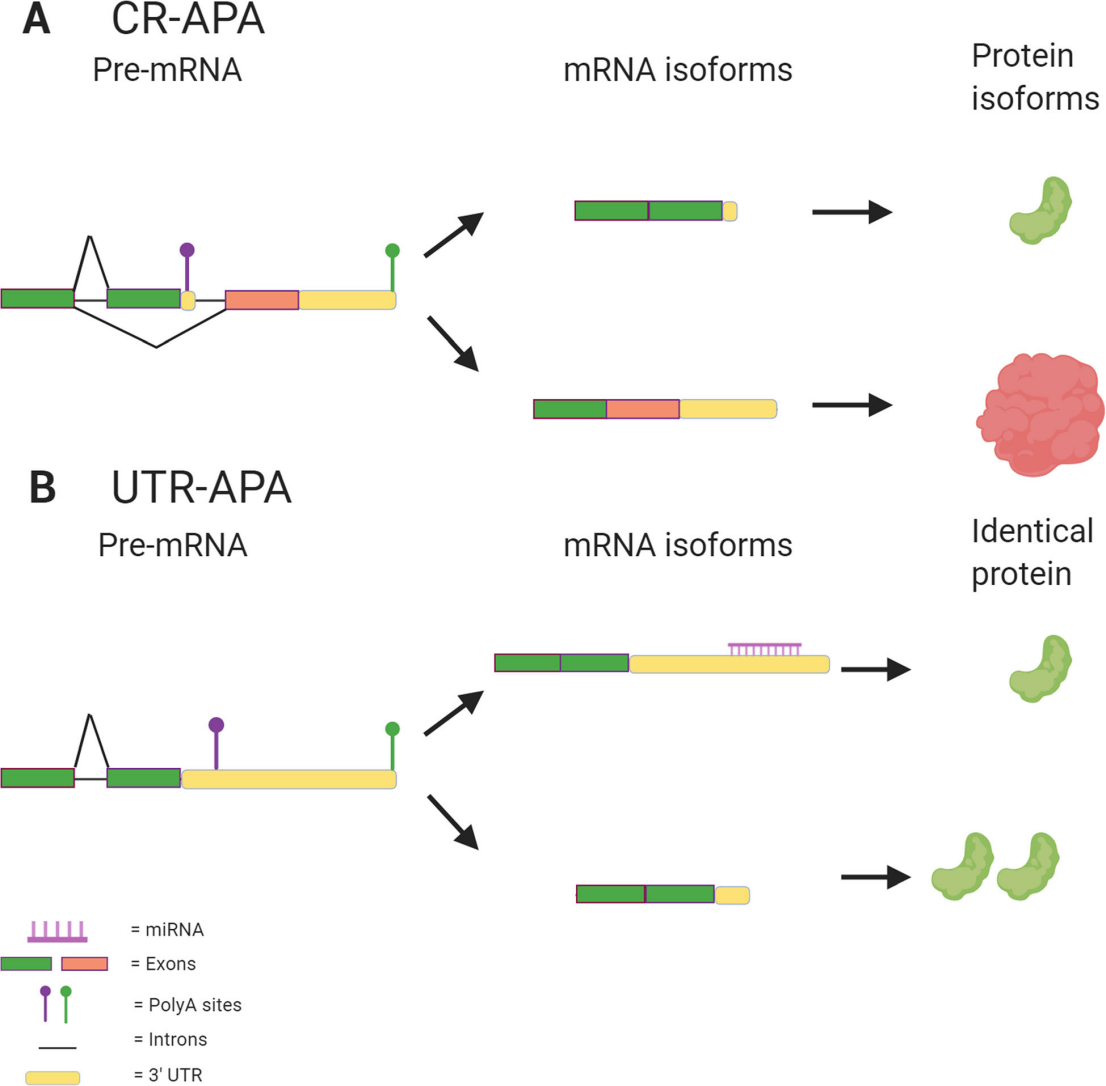
Different variants of APA. **a** In CR-APA the PAS is located within the coding region, which after polyadenylation can result in variations in the coding region at the C-terminal end, resulting in different protein isoforms. Protein isoforms could potentially have different functions within the cell to either lead to or hinder cell proliferation and tumor progression. **b** In UTR-APA, the PAS can be located within the 3′-UTR. Therefore, depending on the location of the PAS, the length of the 3′-UTR could be altered, thus generating new mRNA isoforms whilst not affecting the protein produced. However, the alteration of the 3′-UTR could affect accessibility to regulatory sites, such as for miRNA binding, which would affect expression level of the protein. While not generating new protein isoforms after UTR-APA, oncogenic proteins can be either over or under-expressed as a result of this process

APA is a mechanism that allows a single gene to encode multiple mRNAs, and represents a critical post-transcriptional regulator of gene expression [38]. For the mRNA transcript to undergo APA, a two-step endonucleolytic cleavage of the pre-mRNA occurs at its 3′-UTR, or in some cases within exons and introns of the transcript, followed by addition of repetitive adenosine monophosphate nucleotide units to the end, creating the poly(A) tail [38, 77]. With over 50% of human genes associated with APA, transcripts can be diversified while limiting the size of the genome [38, 78, 119]. These isoform variations are dependent on the location of the alternative poly(A) site (PAS), as some sites can be located within introns or exons, a process known as coding region alternative polyadenylation (CR-APA) (Fig. 1a). This can result in decreased binding of miRNAs to the transcript, as a result of the entire 3′-UTR being cleaved out, in addition to the generation of additional protein isoforms due to the exclusion of exons from the transcript [38, 78, 119]. Another form of APA, termed untranslated region alternative polyadenylation (UTR-APA), occurs when alternative PASs are located in different regions of the 3′-UTR. This results in different 3′-UTR lengths but the same protein isoform, as the coding region remains unaffected (Fig. 1b) [38]. Moreover, previous studies reported that 3′-UTR shortening by APA, by preventing the suppressive effects of miRNAs and other RNA binding proteins, induces the activation of proto-oncogenes [1, 79, 80, 113].

The process of polyadenylation is facilitated by a multimeric protein complex comprised of four primary subunits: the cleavage and polyadenylation specificity factor (CPSF), cleavage stimulation factor (CSTF), mammalian cleavage factor I (CFIm) and cleavage factor II (CFIIm) [18, 40, 120]. CFIm performs a crucial regulatory role in polyA site (PAS) selection by acting as an enhancer-dependent activator [10, 18, 147].

One of the subunits of the CFIm complex, CFIm25, is believed to directly facilitate the recognition of certain PAS sequences, especially those rich in UGUA sequences [79]. Depletion of CFIm25 in GBM leads to 3′-UTR shortening and increased stability of specific transcripts, in turn leading to increased production of oncogenic proteins such as Pak1 and Pak2, key components of the Ras signalling pathway [18]. Activation of the Ras signalling pathway then results in increased cell proliferation and increased GBM aggressiveness [18].

O^6^-methylguanine-DNA methyltransferase (MGMT) is a DNA repair enzyme that acts to convert methylguanine back to guanine by removing the methyl or akyl group from the O^6^ position of guanine, without causing breaks in the DNA. Promoter methylation of the *MGMT* gene in GBM, present in over 40% of cases, results in improved survival in patients treated with TMZ in addition to radiotherapy [45]. APA has been recently identified as an additional mechanism by which MGMT is repressed in GBM. Through the usage of an alternate, distally located PAS of the MGMT transcript, APA results in a transcript variant with an elongated 3′-UTR [60]. This longer 3′-UTR contains miRNA binding sites that act as targets for several miRNAs, including miR-181d, miR-34a, and miR-648, which act to induce degradation of the MGMT transcript [52]. Importantly, this type of promoter-independent silencing of MGMT has also been shown to confer tumor sensitivity to alkylating agents [52, 60]. This concept has important clinical implications, as it should support the use of techniques aiming to identify MGMT protein (e.g. by immunohistochemistry), rather than promoter methylation, to determine MGMT status. However, additional studies are warranted to further elucidate mechanisms of APA in GBM.

## Alternative splicing

**Fig. 2:**
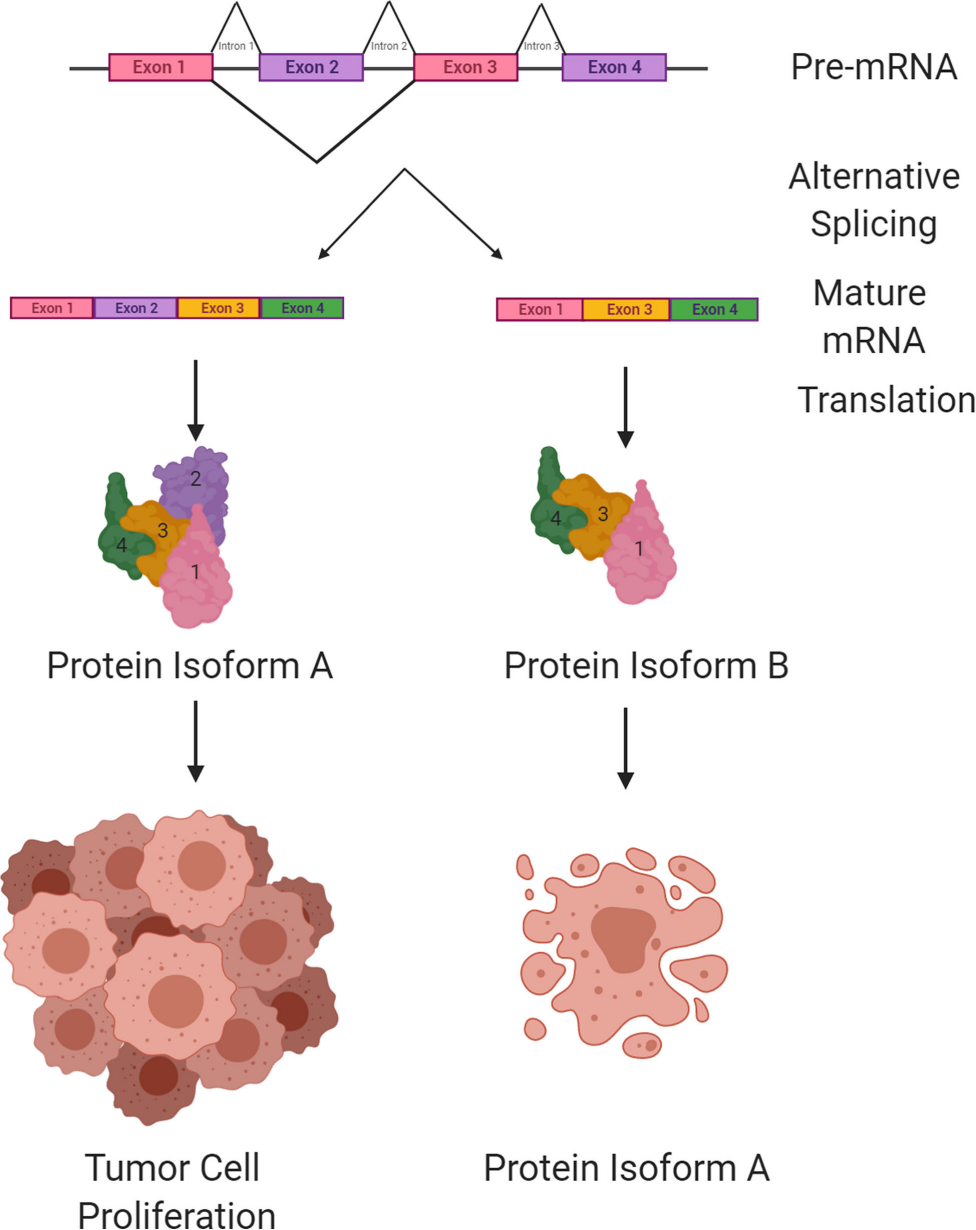
Schematic representation of protein isoforms that can be generated from alternative splicing of pre-mRNA transcript. After removal of the intronic segments of the nascent mRNA transcript by the spliceosome complex (not shown), exons can be ligated to form mature RNA forms that are translated into different protein variants. The different splice variants are implicated in glioma pathogenesis

As mentioned, following transcription, processing of the pre-mRNA transcript precedes downstream translation and protein synthesis. In addition to the modifications discussed above, splicing results in the formation of multiple mRNA and protein isoforms from one gene (Fig. 2). Alternative splicing is performed by excising introns out of a given transcript using a large molecular complex known as the spliceosome, allowing for the synthesis of multiple protein isoforms from one gene [76, 86]. With > 80% of human genes being affected by alternative splicing, the proteome is greatly diversified as a result of the generation of two or more distinct mature mRNA transcripts from each pre-mRNA [47, 116]. By controlling the splice isoforms produced, cells can dynamically change gene expression and favor certain mRNA and protein isoforms to overcome stresses within the microenvironment [85, 91]. The process of splicing is composed of two major steps: the assembly of the spliceosome complex and the actual splicing of the pre-mRNA. The spliceosome is comprised of U1, U2, U4, U5, and U6 small nuclear ribonucleic proteins (snRNPs) and, in the case of the major human spliceosome, it includes over 300 additional proteins [47, 130]. The spliceosome complex is assembled on each target transcript and is directed by specific sequence elements contained within the pre-mRNA, such as the 5′ splice site, the branch point sequence and the 3′ splice site [47, 130]. The mechanistic details behind the cleavage and removal of the spliced out regions of the mRNA have been discussed elsewhere and are beyond the scope of this review [47]. For effective cell adaptation to rapid microenvironmental changes, splicing needs to be as efficient and as precise as possible [76]. However, in reality, pre-mRNA splicing can take up to several hours to be completed, mostly due to varying intron lengths [76]. The precision of splicing is also critical, as a shift in the reading frame and consequent irregularities in splicing can promote the development and progression of different diseases, including cystic fibrosis and glioma [31, 36, 90, 123].

The ubiquitous presence of alternative splicing within cells underscores its relevance for the pathogenesis of tumors, including GBM. Glioma, as described for many other tumors, display a high degree of intertumor heterogeneity [16, 124]. Among the GBM subtypes that can be identified by RNA expression profiling, the mesenchymal (MES) subtype is the most aggressive variant, with higher rates of proliferation in vitro and in vivo and increased radiation resistance [55, 75, 102]. Guardia et al. reported important splicing differences (4934 splicing events affecting 3243 genes) between the MES subtype and the proneural (PN) subtype [43], suggesting a contribution of these events to glioma heterogeneity and plasticity. A large body of literature describes the effects that alternative splicing has on uncontrolled cell proliferation in GBM. The generation of different protein isoforms through alternative splicing promotes increased proliferation and evasion of apoptosis in GBM [118]. Tiek et al. found that different splice variants of Estrogen-related receptor β (ERR-β) influence GBM progression [121]. ERR-β is an orphan nuclear receptor expressed in the brain, where alternative splicing of the 3′ of the pre-mRNA transcript leads to 3 different isoforms: the ERR-β short form (ERR-βsf), ERR-β2, and ERR-β with exon 10 deleted. ERR-β2 drives G2/M cell cycle arrest and induces apoptosis [44]. Exploring ERR-β2 function in GBM, these authors found that by favoring expression of ERR-β2 over other splice variants and by inhibiting the splicing regulatory cdc2-like kinases (CLKs), they could suppress GBM cell migration and proliferation, in combination with an ERR-β2 agonist [121].

The MAPK interacting kinase (Mnks) family of proteins include MNK1 and MNK2 and are the kinases responsible for phosphorylation of eukaryotic translation initiation factor 4E (eIF4E) on Ser-209 [56]. While genetic alterations of the *MKNK1* and *MKNK2* genes are rare in cancer, being present in 0.5 and 2.4% of cases respectively in glioma [13], both proteins undergo alternative splicing to create distinct protein isoforms [84]. Previous studies have suggested that MNK1 positively regulates the expression of TGFβ, known to regulate proliferation, invasion and immune evasion [4, 41, 42]. A recent study by Garcia-Recio et al. reported that the MNK1b isoform (the spliced variant of MNK1a lacking the 89 C-terminal amino acids [42]) can act as a marker for prognosis in breast cancer patients [37]. Unfortunately, the clinical outcomes resulting from the formation of Mnk1a and Mnk1b have not been analyzed in detail with regard to brain tumors. Mnk2b, acts as an oncogenic protein by inducing eIF4E phosphorylation and not activating p38-MAPK, leading to enhanced translation of mRNAs encoding factors implicated in tumor formation, such as c-MYC and cyclin D1 [94]. On the other hand, one of the MNK2 isoforms, Mnk2a, acts as a tumor suppressor by co-localizing with and activating the p38-MAPK pathway to induce apoptosis and suppressing Ras-induced transformation [73]. Typically, the p38-MAPK pathway is activated in response to environmental changes and affects transcription, gene expression and efficacy of drug therapies [5, 59, 66]. The mechanism of action of many anticancer drugs has been linked to induction of the p38-MAPK pathway. Thus, one potential therapeutic strategy could rely on utilizing drugs to favor the Mnk2a isoform, which can activate the p38-MAPK stress response more effectively and promote apoptosis [103, 133]. As a specific pre-clinical example in GBM, Mogilevsky et al. recently used splice switching oligonucleotides (SSOs) that bind to Mnkb2 splice sites on the MKNK2 pre-mRNA to disrupt normal splicing by blocking interactions between the pre-mRNA and the spliceosome. This resulted in suppression of Mnk2b production and GBM growth in-vivo, and re-sensitized cells to chemotherapy [75]. Finally, a recent article reported non-coding mutations in the 5′ splice site binding region in U1 spliceosomal small nuclear RNAs (snRNAs), resulting in increased 5′ cryptic splicing events and aberrant RNA splicing in Sonic hedgehog (SHH) medulloblastoma, compared to control cells [114]. This results in the inactivation of tumor suppressing genes such as *PTCH1* while activating oncogenic genes such as *GLI2* and *CCND2* [114]. While no literature to date has investigated snRNA mutations in GBM, these studies emphasize how controlling the regulation of alternative splicing to favour the production of tumor suppressive isoforms has potential for developing novel therapeutic approaches for GBM.

## Adenosine to inosine modifications

**Fig. 3:**
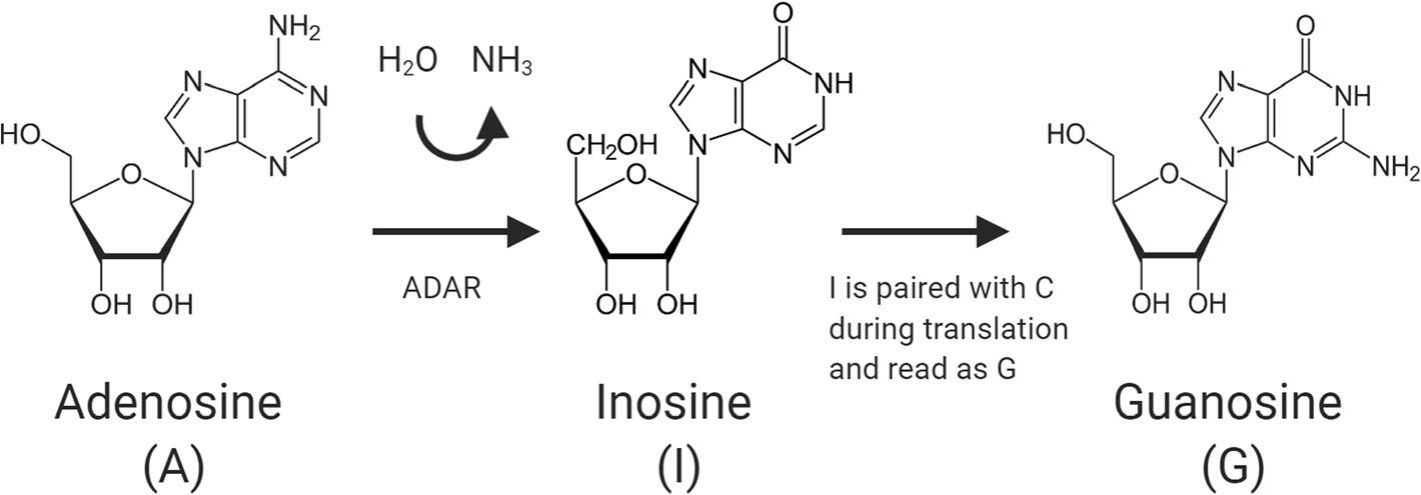
The hydrolytic deamination of adenosine is catalyzed by ADAR enzymes, resulting in the formation of inosine. Due to structural similarities between inosine and guanosine, the translational machinery reads the nucleoside as the latter, potentially resulting in changes to codon sequences or the addition/removal of splice sites. The A-to-I modification can also serve to direct alternative splicing to create oncogenic protein isoforms that promote glioma progression

A-to-I modifications comprise the irreversible, hydrolytic deamination of adenosine nucleoside bases by adenosine deaminases acting on dsRNA (ADARs), converting the adenosine to inosine in RNAs [3, 87, 98, 106]. Inosines are then recognized by the translation machinery as guanosines rather than adenosines, due to preferential base pairing of inosine with cytidine (Fig. 3) [26, 97, 122]. This substitution can lead to codon changes, modify the amino acid sequence of proteins, change the secondary structure of RNA, and result in the addition or removal of splice sites to expand the proteome [2, 87, 93]. In mammals, three enzymes that catalyze A-to-I modifications have been identified to date: ADAR1, ADAR2 and ADAR3 [88]. A-to-I modifications can also affect processes such as the binding of miRNAs to 3′-UTRs through editing of the miRNA *seed* sequence (the miRNA recognition site in RNAs), affecting target specificity of the mature miRNA [32, 57, 137]. A-to-I modifications are even responsible for directing the alternative splicing of its own pre-mRNA with the ADAR2 enzyme, resulting in multiple isoforms with different catalytic functions each [34].

While found in all human tissues, A-to-I editing is most prevalent in the brain, and aberrant editing is linked to the development of various brain pathologies including amyotrophic lateral sclerosis, Alzheimer’s disease, Parkinson’s disease, epilepsy and glioma [14, 34, 93, 107]. A-to-I editing is extremely prevalent in healthy brains, with almost 100% of some miRNA strands being edited, such as in the case of miR-589-3p, resulting in inhibition of aberrant cell proliferation [14]. In GBM, while mutations or deletions of the *ADARB1* gene occur only in 0.6% of cases [13], ADAR2 activity is decreased, leading to significant hypo-editing of miRNAs, resulting in miRNA target switching by changing the miRNA *seed* sequence [14]. In the case of miR-589-3p, this is retargeted from tumor-suppressor Protocadherin 9 (PCDH9) to Disintegrin and Metalloproteinase 12 (ADAM12), facilitating GBM invasion by supporting cell adhesion [14, 58, 74].

GBM is known to utilize glutamate to promote proliferation and migration [49], a process mostly mediated by calcium permeable AMPA-type glutamate receptor signaling. One of the subunits, glutamate receptor subunit B (GluR-B), encoded by GRIA2, was one of the first known ADAR targets [71, 138]. By editing a single adenosine on the GRIA2 transcript, known as the glutamine/arginine (Q/R) site targeted by ADAR2 [72], the originally encoded Q codon is converted to positively charged R encoding codon, allowing for the incorporation of the GluR-B subunit into the AMPA receptor, rendering it impermeable to positively charged calcium ions [132]. If the transcript is left unedited due to a reduction in ADAR activity, such as in GBM, AMPA remains permeable to calcium. This results in increased activity of AMPA-type glutamate receptors independently from glutamate activation, leading to excitotoxicity and epileptic seizures, typically associated with glioma [48, 72, 88]. However, observed changes in ADAR2 activity are not due to decreased expression of ADAR2, but rather to inhibition of self-editing leading to decreased alternative splicing of the ADAR2 pre-mRNA transcript [72]. Q/R site editing of GRIA2 is edited in virtually 100% of healthy mammalian brain tissue [109]. In contrast, Maas et al. found that Q/R editing decreases to 90% in lower grade astrocytoma and decreases further to 69–88% in GBM [72]. Unfortunately, the cellular mechanisms that induce these changes and regulate ADAR2 activity in brain tumors remain unclear [72]. Thus, an emphasis on targeting the self-editing site to further increase editing at that location, in combination with increasing expression of ADAR2, should be the focus of further pharmacological research.

In other studies, the activity of ADAR2 was deemed essential to prevent glioma proliferation and growth through the editing of the CDC14B pre-mRNA transcript involved in the Skp2/p21/p27 pathway [35]. As ADAR2 activity decreases in gliomas, CDC14B pre-mRNA modification is decreased, resulting in overexpression of Skp2 and downregulation of the known tumor suppressors p21 and p27 [35]. This eventually induces glioma cells to bypass the G1/S checkpoint, promoting increased cell proliferation [35]. Oakes et al. found that as a negative regulator of ADAR2 activity, ADAR3, which is genetically amplified in ~ 2% of glioma, inhibits the binding of ADAR2 to the GRIA2 pre-mRNA transcript, preventing RNA editing [88], although the exact mechanism by which ADAR3 performs this function remains unclear. Without the deaminase activity of ADAR1 and ADAR2 in vitro or in vivo*,* it is unlikely that ADAR3 can directly edit pre-mRNA transcripts. However, as ADAR3 is known to bind to dsRNA despite not being able to perform A-to-I modifications, it has been hypothesized that ADAR3 could act as a direct physical block, preventing ADAR2 from mRNA binding and subsequent editing [88]. An alternative scheme is that ADAR3 facilitates the alternative splicing of the GRIA2 pre-mRNA transcript, hence providing another method of preventing ADAR2 editing to promote glioma proliferation and malignant progression [88]. Sequestering ADAR3 or upregulating ADAR2 would serve as potential therapeutic strategies that could increase pre-mRNA transcript editing, decreasing GBM progression.

## Conclusions

The studies outlined in this review highlight the importance of RNA modifications in brain tumor progression, specifically in glioma. The flexibility conferred by post-transcriptional control adds another dimension by which gene expression can be regulated beyond what is directly coded from DNA. The addition of multiple modifications on the same transcript could thus increase the complexity of multiple regulatory networks. This plasticity is particularly relevant for cancer cells to adapt to unexpected microenvironmental changes. However, as showcased throughout this review, deregulation of the RNA modification machinery and altered gene expression are associated with many of the hallmarks of cancer, such as apoptosis evasion and uncontrolled proliferation. Given the significant contribution to brain tumor malignancy, greater emphasis on clarifying the role of RNA regulation and modifications in glioma progression is needed. Targeting the regulatory enzymes controlling the post-transcriptional modifications discussed in this paper warrants further detailed investigation, as this remains an unexplored strategy that could ultimately improve the prognosis of brain tumor patients.

## Data Availability

Not applicable.

## Abbreviations

3′- UTR: 3′ - Untranslated region
AML: Acute myeloid leukemia
ADAM12: Disintegrin and Metalloproteinase 12
ADAR: Adenosine deaminases acting on dsRNA
ALKBH5: AlkB Homolog 5
APA: Alternative polyadenylation
CFIIm: Mammalian cleavage factor II
CFIm: Mammalian cleavage factor I
CLK: Cdc2-like kinases
CPSF: Cleavage and polyadenylation specificity factor
CSTF: Cleavage stimulation factor
DOT1L: Disrupter of telomeric silencing 1-like
EGFR: Epidermal growth factor receptor
eIF4E: Eukaryotic translation initiation factor 4E
ERR-β: Estrogen-related receptor β
ERR-βsf: ERR-β short form
FTO: Fat mass and obesity-associated protein
GBM: Glioblastoma
GO: Gene Ontology
GSEA: Gene set enrichment analysis
GluR: Glutamate receptor
HIF: Hypoxia-inducible factors
m^6^A: N^6^-methyladenosine
MeRIP-seq: Methylated RNA immunoprecipitation-seq
MES: Mesenchymal
METTL14: Methyltransferase-like 14
METTL3: Methyltransferase-like 3
MGMT: O6-methylguanine-DNA-methyltransferase
miRNA: Micro RNA
NGS: Next generation sequencing
NMD: Nonsense-mediated mRNA decay
PAS: Poly(A) site
PAR-CLIP: Photoactivatable ribonucleoside-enhanced crosslinking and immunoprecipitation
PCDH9: Protocadherin 9
PN: Proneural
QKI: Quaking I
RIP-seq: RNA immunoprecipitation sequencing
SgRNA: Guide RNA
ShRNA: Short hairpin RNA
snRNA: Small nuclear RNA
SRSF: Serine and arginine rich splicing factor
SSO: Splice switching oligonucleotides
TCGA: The Cancer Genome Atlas
TMZ: Temozolomide
WTAP: Wilms’ tumor 1-associating protein
YTH: YT521-B homology
YTHDC: YTH domain containing
YTHDF: YTH N^6^-methyladenosine RNA binding protein
